# Subjective cognitive impairment and brain structural networks in Chinese gynaecological cancer survivors compared with age-matched controls: a cross-sectional study

**DOI:** 10.1186/s12885-017-3793-4

**Published:** 2017-11-28

**Authors:** Yingchun Zeng, Andy S. K. Cheng, Ting Song, Xiujie Sheng, Yang Zhang, Xiangyu Liu, Chetwyn C. H. Chan

**Affiliations:** 1 Department of Rehabilitation Sciences, The Hong Kong Polytechnic University, Hung Hom, Hong Kong, China; 2 Research Institute of Gynecology and Obstetrics, The Third Affiliated Hospital of Guangzhou Medical University, Guangzhou, China; 3 Department of Radiology, The Third Affiliated Hospital of Guangzhou Medical University, Guangzhou, China; 4 School of Nursing, Guangzhou Medical University, Guangzhou, China; 5 Department of Nursing, Hunan Cancer Hospital, The Third Affiliated Cancer Hospital of Xiangya School of Medicine, Central South University, Changsha, China

**Keywords:** Subjective cognitive impairment, Chemotherapy, Brain networks, Gynaecological cancer, China

## Abstract

**Background:**

Subjective cognitive impairment can be a significant and prevalent problem for gynaecological cancer survivors. The aims of this study were to assess subjective cognitive functioning in gynaecological cancer survivors after primary cancer treatment, and to investigate the impact of cancer treatment on brain structural networks and its association with subjective cognitive impairment.

**Methods:**

This was a cross-sectional survey using a self-reported questionnaire by the Functional Assessment of Cancer Therapy-Cognitive Function (FACT-Cog) to assess subjective cognitive functioning, and applying DTI (diffusion tensor imaging) and graph theoretical analyses to investigate brain structural networks after primary cancer treatment.

**Results:**

A total of 158 patients with gynaecological cancer (mean age, 45.86 years) and 130 age-matched non-cancer controls (mean age, 44.55 years) were assessed. Patients reported significantly greater subjective cognitive functioning on the FACT-Cog total score and two subscales of perceived cognitive impairment and perceived cognitive ability (all *p* values <0.001). Compared with patients who had received surgery only and non-cancer controls, patients treated with chemotherapy indicated the most altered global brain structural networks, especially in one of properties of small-worldness (*p* = 0.004). Reduced small-worldness was significantly associated with a lower FACT-Cog total score (*r* = 0.412, *p* = 0.024). Increased characteristic path length was also significantly associated with more subjective cognitive impairment (*r* = −0.388, *p* = 0.034).

**Conclusion:**

When compared with non-cancer controls, a considerable proportion of gynaecological cancer survivors may exhibit subjective cognitive impairment. This study provides the first evidence of brain structural network alteration in gynaecological cancer patients at post-treatment, and offers novel insights regarding the possible neurobiological mechanism of cancer-related cognitive impairment (CRCI) in gynaecological cancer patients. As primary cancer treatment can result in a more random organisation of structural brain networks, this may reduce brain functional specificity and segregation, and have implications for cognitive impairment. Future prospective and longitudinal studies are needed to build upon the study findings in order to assess potentially relevant clinical and psychosocial variables and brain network measures, so as to more accurately understand the specific risk factors related to subjective cognitive impairment in the gynaecological cancer population. Such knowledge could inform the development of appropriate treatment and rehabilitation efforts to ameliorate cognitive impairment in gynaecological cancer survivors.

## Background

Cognitive impairment can be a significant and prevalent problem for survivors with gynaecological cancer [1, 2]. Cognitive impairment often refers to cancer-related cognitive impairment (CRCI), which can be related to the cancer itself, as well as to its treatment, for example surgery, chemotherapy, and radiation therapy [3, 4]. As CRCI can negatively impact quality of life and daily life functioning in cancer survivors [5, 6], it is important to investigate such late effects and to understand the course and causes of CRCI for guiding future treatment and rehabilitation efforts [7–10].

CRCI may be related to a number of psychological factors that are seldom investigated in the context of gynaecological cancer, although gynaecological cancer is the second most prevalent form of cancer among women in China [11]. Psychological distress has been found to be negatively associated with neuropsychological performance in cancer patients [8, 12]. Research has also found that perceived cancer-related fatigue and anxiety resulted in CRCI in cancer survivors [13, 14]. Furthermore, treatment-related mood changes, such as depression, have also significantly influenced many cancer survivors’ cognitive functioning [13, 15–17]. Other research found that age and education levels were also associated with changes in cognitive function among gynaecological cancer survivors [6].

Self-reported cognitive impairment are also associated with structural and functional changes in the brain that can be detected by magnetic resonance imaging (MRI) [10, 17]. Brain networks are organised such that specialised regions or clusters of neurons are highly connected to their neighbours but sparsely connected to distant regions [18]. “Brain structural network that tends to display as a small-world network organization, which is characterized by high clustering in local regions while retaining relatively short path lengths across all brain regions, supporting the notion of the brain in an optimal balance between segregation and integration in information processing between brain regions [10, 19, 20]”. “Cognitive functions are believed to be supported by parallel neural networks that must balance the competing demands of segregation and integration [21].” Observational studies have found that alterations in the brain structural network have been demonstrated to have adverse effects on cognition in both female and male cancer survivors [10, 22]. However, much remains unknown about the effects of cancer and its treatment on brain structural networks in gynaecological cancer populations.

In view of the poor understanding of the psychosocial impacts of cancer treatment on perceived cognitive impairment and brain networks in gynaecological cancer survivors, it is important to explore CRCI and its associated factors in this population. Therefore, this study aims to assess subjective cognitive functioning in gynaecological cancer survivors after primary cancer treatment, and to investigate the impact of cancer treatment on brain structural networks and cancer treatment’s association with cognitive impairment. This study also aims to explore associated predictors of subjective cognitive impairment in order to gain a deeper understanding of potential factors of importance to CRCI.

## Methods

This cross-sectional study was conducted to assess research participants’ subjective cognitive functioning, psychological wellbeing and brain structural networks immediately after primary cancer treatment. Age-matched non-cancer controls were simultaneously assessed using these outcome measures for direct comparison.

### Subjects

Patients were recruited in South China, at a tumor hospital and at a general hospital’s gynaecological oncology unit. This study obtained ethical approval from the ethics committees at both Hunan Cancer Hospital and The Third Affiliated Hospital of Guangzhou Medical University. Subjects were Chinese females ages 18 to 60 years; with a primary diagnosis of gynaecological cancer, without metastatic disease. Patient exclusion criteria were women who did not have a primary diagnosis of cancer, and/or who were in a terminal stage of cancer. Advertisements to recruit non-cancer controls were posted in the hospitals’ common areas. Each non-cancer control subject was within 2 years of the age of the patient. All participants had to be without a diagnosis of a neurodegenerative disease or any potential psychiatric disorder, and without the use of psychotropic medication. All study participants provided written informed consent to participate.

### Self-reported cognitive measures

Subjective cognitive functioning was assessed using the Functional Assessment of Cancer Therapy-Cognitive (FACT-Cog) scale. A self-report questionnaire measures perceived cognitive impairment, comments from others, perceived cognitive ability, and impact of cognitive impairment on quality of life [23]. The FACT-Cog consists of 37 items and the overall cognitive function is the sum of the four subscales [23]. Higher scores indicate better cognitive function (i.e. lower subjective cognitive impairment).

### Psychological measures and general information sheet

Depression and anxiety were evaluated using the Chinese version of the Hospital Anxiety and Depression Scale (HADS) [24]. The HADS is a 14-item self-assessment scale for a screening instrument to assess patients’ anxiety and depression levels. Each item is scored from 0 to 3. The anxiety and depression sub-scores are both on scales of 0 to 21. Higher total scores indicate higher levels of anxiety and depression [24]. The Chinese version of HADS has been reported to have acceptable internal consistency and validity [25, 26], and is found to be a reliable tool for assessing psychological disturbances in cancer survivors [24]. The Brief Fatigue Inventory (BFI) has been validated as a short and comprehensive instrument to assess the severity of fatigue and fatigue-related impairment in cancer survivors [27, 28]. It consists of 10 items and allows a basic assessment of the dimensions of activity, ability to walk, mood, work, interpersonal relationships, and enjoyment of life [27]. Lower scores indicate less severity of fatigue [27]. A general information sheet collected subjects’ demographic and clinical characteristics in terms of age, education level, employment, and marital status. Patients’ clinical information included cancer types, disease stage, and treatment received (e.g., Surgery, Radiation, Chemotherapy).

### MRI data acquisition

The MRI data were acquired using a Philips 3 T Achieva MRI scanner with an 8-channel head coil. Structural brain networks were assessed in 30 participants who reported cognitive complaints (with the FACT-Cog summary score ≤ 85): ten out of 130 healthy controls had a FACT-Cog summary score of less than 85, then 10 age-matched surgery patients and 10 age-matched patients with chemotherapy who also reported cognitive complaints were included in brain MRI scanning.. This cut-off score for the FACT-Cog was based on existing published studies [17, 29]. DTI (diffusion tensor imaging) and high-resolution structural T1-weighted brain scans were obtained using single-shot echo-planar imaging (EPI) (acquisition matrix = 128 × 128; TE = Minimum; TR = 16,000 ms; field of view = 256 mm × 256 mm; slice thickness/gap = 2.0 mm/0 mm; scanning time = 6 min 56 s) with 32 distributed isotropic orientations for the diffusion-sensitising gradients at a b-value of 1000 s/mm^2^and a b-value of 0. T1-weighted imaging was achieved for morphometric (GM volume, cortical thickness and surface area) analysis using three-dimensional fast spoiled-gradient recalled acquisition in steady state in 166 coronal slices (acquisition matrix = 128 × 128; TE = 3.9 ms; TR = 9.6 ms; field of view = 256 mm × 256 mm; slice thickness/gap = 2 mm/0 mm; scanning time approximately 7 min).

### Statistical analysis

Data related to self-reported cognitive and psychological measures were analysed using SPSS for Windows (version 21; IBM SPSS Statistics, Armonk, NY, USA). Descriptive, comparison and regression analysis were used to analyse behavioural data. Descriptive statistics were used to describe sociodemographic and clinical characteristics of the sample. In comparing subject characteristics, cognitive and psychological measures between patients and healthy controls used independent t-tests for continuous variables, and chi-square or Fischer exact tests were used for testing differences in categorical measures. To analyse the relationship between perceived cognitive functioning and associated factors, a univariate analysis was used followed by multiple regression analysis. As sociodemographic factors, such as age and education level, have been found to be associated with cognitive functioning in ovarian cancer survivors [6], it is important to adjust for these factors, as they may potentially confound the relationship between subjective cognitive impairment and associated factors. The residuals’ normality, linearity, and homoscedasticity were checked to ensure the validity of the linear regression model [30]. Multiple linear regression analysis was performed using a forward stepwise approach, in which variables were significantly related to the total FACT-Cog score in the univariate analysis (it must be at a cutoff *p* value of 0.1 in the univariate analysis). Analysis of variance (ANOVA) was used to compare three participant groups reported with subjective cognitive impairment and brain structural network properties. Associations between brain network properties and subjective cognitive impairment were explored using Pearson’s correlation coefficient. The threshold of *P* < 0.05 was used to assess statistical significance.

### MRI data processing and analyses

The DTI images were preprocessed using PANDA: a pipeline toolbox for analysing brain diffusion images (https://www.nitrc.org/projects/panda/). Each individual’s DTI dataset was registered to the same individual’s high-resolution structural image and then into the standard Montreal Neurological Institute (MNI) space using affine transformations. Fractional Anisotropy (FA) images were created from the pre-processed DTI data of all subjects. All FA images were then non-linearly aligned to a common space. The mean FA image was used to represent the centre of all tracts common to the group. Then, all subjects’ aligned FA data were projected onto the skeleton, and the resulting data were subjected to voxelwise cross-subject statistics. Whole brain tractography was then performed in the patient’s native space for each subject at each time point using a deterministic streamlined approach [31, 32], in which fibre pathways were reconstructed by following the main diffusion tensor direction as indicated by the principal eigenvector, until an FA value of 0.20 or lower was reached, or until an angular turn of 45 degrees or more was made [31, 32].

The assessment of brain network measures was performed using the toolkit of graph theoretical network analysis (GRETNA) (https://www.nitrc.org/projects/gretna/). The voxelwise brain structural network analyses were performed using the CPU-GUI platform of GRETNA [33]. The following characteristic graph metrics were estimated to describe the topological organisation of the whole brain structural networks: global topological properties consist of small-world measures and global network efficiency; local topological properties include local network efficiency, nodal clustering coefficient, and nodal shortest path length.

## Results

### Research participant characteristics

Of the 288 participants, 158 patients with gynaecological cancer had completed primary cancer treatment within a week, and 130 noncancer controls were balanced in terms of age and marital status (Table 1). Nearly half of patient participants (*n* = 81, 51.3%) were in the early stages of cancer, more than 60% of patients (*n* = 98, 62.0%) had a diagnosis of cervical cancer, and more than half of patients were receiving chemotherapy or a combination of chemotherapy and other cancer treatment. All research subjects’ demographic and clinical characteristics are shown in Table 1.

**Table 1:** Demographic and clinical characteristics of participant groups

Variables	Mean (SD) / n (%)		
	Patients (*n* = 158)	Healthy controls (*n* = 130)	*p*-Value
Age (years)	45.86 (10.56)	44.55 (9.72)	0.157
Education levels			<0.001
Primary school or below	103 (65.2)	65 (50.0)	
High school	34 (21.5)	15 (11.5)	
College or above	21 (13.3)	50 (38.5)	
Employment status			<0.001
Employed but on medical leave	32 (20.3)	100 (76.9)	
Unemployed or retired	126 (79.7)	30 (23.1)	
Marital status			0.895
Single	9 (5.7)	8 (6.2)	
Married	142 (89.9)	117 (90.0)	
Divorced	6 (3.8)	5 (3.8)	
Widowed	1 (0.6)	0 (0.0)	
Disease stage			
Early stage	81 (51.3)		
Middle stage	57 (36.1)		
Advanced stage	20 (12.6)		
Disease diagnosis			
Cervical cancer	98 (62.0)		
Ovarian cancer	28 (17.7)		
Uterine cancer	14 (8.9)		
Other (e.g. GTN)	18 (11.4)		
Types of treatment			
Surgery	37 (23.4)		
Chemotherapy	14 (8.9)		
Surgery + chemotherapy	71 (44.9)		
Surgery + radiation + chemotherapy	21 (13.3)		
Radiation + chemotherapy	15 (9.5)		

*Abbreviation: GTN* Gestational Trophoblastic Neoplasia

### Self-reported cognitive functioning

From Table 2, gynaecological cancer survivors reported statistically higher subjective cognitive impairment than healthy controls (*p* < 0.001), especially in the subscale scores of perceived cognitive impairment and perceived cognitive ability (all *P* values <0.001). Within the patient group, patients receiving chemotherapy scored lower in the FACT-Cog total scores and four subscale scores (Table 3). According to Vardy et al. [17, 29], subjects were categorised as having subjective cognitive impairment with a FACT-Cog score of 85 or less. Of 158 patients, a total of 64 (40.51%) reported subjective cognitive impairment. Within the patient group, 16 subjects (10.13%) in the surgery group had subjective cognitive impairment and 48 subjects (30.38%) receiving chemotherapy had perceived cognitive impairment. Of 130 healthy controls, a total of 10 subjects (7.69%) perceived cognitive impairment.

**Table 2:** Mean scores of cognitive and psychological measures in each group

Measures	Mean (SD)		*p*-Value
	Patient (*n* = 158)	Healthy controls (*n* = 130)	
FACT-Cog	99.80 (19.67)	112.33 (21.53)	**<0.001**
Perceived cognitive impairment	57.38 (11.51)	65.21 (12.20)	**<0.001**
Comments from others	14.51 (2.75)	14.37 (2.26)	0.658
Perceived cognitive ability	16.24 (6.81)	21.20 (8.02)	**<0.001**
Impact on QOL	11.53 (3.54)	11.73 (4.05)	0.667
HADS			
Anxiety	5.86 (4.28)	5.40 (3.86)	0.352
Depression	5.17 (4.33)	4.52 (3.50)	0.171
BFI-total	32.52 (21.20)	27.25 (21.72)	0.062

*Abbreviation: BFI* Brief Fatigue Inventory, *FACT-Cog* Functional Assessment of Cancer Therapy-Cognition, *HADS* Hospital Anxiety and Depression Scale

Bolded *p* values are statistically signifcant

**Table 3:** Mean scores of cognitive and psychological measures in the patient group

Measures	Mean (SD)		*p*-Value
	Surgery only (*n* = 37)	Receiving CT (*n* = 121)	
FACT-Cog	108.25 (17.84)	97.28 (19.55)	**0.003**
Perceived cognitive impairment	61.76 (8.74)	56.04 (11.95)	**0.002**
Comments from others	15.24 (1.77)	14.29 (2.95)	**0.019**
Perceived cognitive ability	18.22 (7.71)	15.66 (6.44)	**0.047**
Impact on QOL	13.21 (3.12)	11.28 (4.20)	**0.003**
HADS			
Anxiety	4.35 (3.97)	6.32 (4.28)	**0.014**
Depression	4.00 (3.28)	5.52 (4.31)	0.060
BFI-total	19.08 (17.41)	34.33 (22.26)	**<0.001**

*Abbreviation: BFI* Brief Fatigue Inventory, *CT* Chemothrapy, *FACT-Cog* Functional Assessment of Cancer Therapy-Cognition, *HADS* Hospital Anxiety and Depression Scale

Bolded *p* values are statistically signifcant

### Psychological measures and associated predictors of FACT-cog outcomes

While there were no statistically significant differences between patients and healthy controls in terms of anxiety, depression, and fatigue levels (Table 2), there were greater anxiety and fatigue levels in the subgroup of patients receiving chemotherapy (*p* = 0.014, and *p* < 0.001, respectively) (Table 3). Associated predictors of cognitive outcomes were explored using multivariate linear regression analysis (Table 4). Hierarchical multiple regression analyses were performed to identify significant associated factors for subjective cognitive impairment. A forward stepwise approach was used. There were no confounders accounting for the association between associated predictors and perceived cognitive functioning. The total variance explained by the linear regression model was 40.8%. Employment status, receipt of chemotherapy and depressive symptoms were statistically significant predictors of perceived cognitive functioning (standardized beta = −0.199, −0.129 and −0.331, respectively; all *p* values <0.05).

**Table 4:** Factors associated with cognitive complaints (FACT-Cog) in the patient group

Variables	Unstandardized Coefficients (B)	Standard Error	Standardized Coefficients Beta	*p*-Value
Age	−0.129	0.123	−0.070	0.294
Employment status	−9.884	3.336	−0.199	**0.004**
Receipt of chemotherapy	−6.077	2.997	−0.129	**0.044**
Anxiety	−0.935	0.515	−0.203	0.071
Depression	−1.503	0.488	−0.331	**0.002**
BFI total score	−0.127	0.065	−0.140	0.055

*Abbreviation: BFI* Brief Fatigue Inventory, *FACT-Cog* Functional Assessment of Cancer Therapy-Cognition

Adjusted *R* ^2^ = 0.408, *p* < 0.001

Bolded *p* values are statistically signifcant

### Brain structural networks and correlations with subjective cognitive impairment

**Fig. 1:**
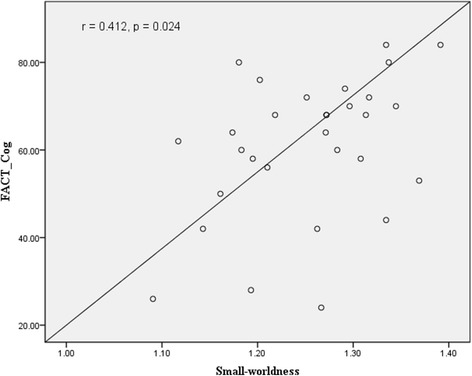
Correlation of small-worldness properties with FACT-Cog total score

**Fig. 2:**
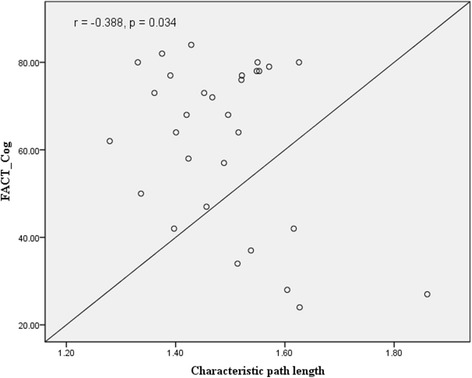
Correlation of characteristic path length with FACT-Cog total score

From Table 5, within global topological properties three groups had a small-world connectome organisation, as the mean small-worldness index was greater than one. There were statistically significant differences in terms of small-worldness index (*p* = 0.004). Patients receiving chemotherapy had the lowest mean small-worldness index, compared with patients who received surgery only and healthy controls. Lower small-worldness index was associated with more subjective cognitive impairment (*r* = 0.412, *p* = 0.024) (Fig. 1). For the local topological properties, there were no statistically significant differences including nodal efficiency, nodal clustering coefficient, and shortest path length (all *p* values >0.05). Shorter characteristic path length, which indicates more efficient network organisation, was significantly associated with fewer subjective cognitive impairment (*r* = −0.388, *p* = 0.034) (Fig. 2).

**Table 5:** Demographics, cognitive function and brain network measures in each group

	Mean (SD)/ n (%)			*p*-Value
	Surgery only (*n* = 10)	Receiving CT (*n* = 10)	Healthy controls (*n* = 10)	
Age	50.50 (9.51)	50.90 (9.34)	50.50 (6.81)	0.993
Education levels				0.159
Primary school or below	8 (80.0)	9 (90.0)	7 (70.0)	
High school or above	2 (20.0)	1 (10.0)	3 (30.0)	
Employment status				0.329
Employed	1 (10.0)	0 (0)	10 (100)	
Unemployed or retired	9 (90.0)	10 (100)	0 (0)	
Marital status				0.355
Married	10 (100)	9 (90.0)	10 (100)	
Divorced	0 (0)	1 (10.0)	0 (0)	
FACT-Cog total score	77.50 (6.00)	59.40 (4.85)	78.60 (5.81)	**<0.001**
Perceived cognitive impairment	43.70 (3.83)	31.90 (11.21)	46.50 (5.68)	**0.046**
Comments from others	13.90 (2.76)	9.30 (4.94)	11.60 (3.68)	0.070
Perceived cognitive ability	12.10 (2.02)	9.70 (4.69)	13.60 (3.65)	0.687
Impact on QOL	7.80 (3.64)	8.50 (4.19)	6.90 (4.45)	**<0.001**
Graph metrics				
Small-worldness	1.276 (0.039)	1.191 (0.074)	1.290 (0.073)	**0.004**
Global efficiency	0.140 (0.002)	0.136 (0.004)	0.142 (0.005)	0.065
Local efficiency	0.199 (0.004)	0.199 (0.005)	0.201 (0.004)	0.697
Clustering coefficient	1.696 (0.296)	1.765 (0.314)	1.504 (0.261)	0.137
Characteristic path length	1.487 (0.099)	1.544 (0.139)	1.446 (0.094)	0.169

*Abbreviation: CT* Chemothrapy

Bolded *p* values are statistically signifcant

## Discussion

This was a cross-sectional survey using a self-reported questionnaire to assess subjective cognitive functioning, and applying DTI and graph theoretical analyses to investigate brain structural networks after primary cancer treatment. Compared with non-cancer controls, patients reported a higher prevalence of subjective cognitive impairment, especially in the subgroup of patients receiving chemotherapy. Regression analysis also confirmed that receipt of chemotherapy was one of the significant predictors of CRCI. Other risk factors related to CRCI in gynaecological cancer survivors included employment status and depression. Consistent with previous studies, the prevalence of CRCI was significantly higher in survivors with depression than in survivors without depression [15, 16, 34]. Yet at the same time, in the non-depressed survivors, the severity CRCI was significantly higher in survivors receiving chemotherapy than in survivors without receipt of chemotherapy. This study finding suggests that CRCI could possibly be associated with chemotherapy rather than depressive symptoms. Previous research found that Chinese female cancer survivors reported higher levels of anxiety and depression, resulting in lower levels of work productivity [35]. In consequence, employed cancer survivors experienced work limitations more frequently, leading to more cognitive impairment.

While there is a growing concern regarding possible CRCI following primary cancer treatment [36], appropriately assessing cognitive impairment in cancer survivors is an important aspect of CRCI [37]. “CRCI is usually subtle, and standard definitions of impairment on neuropsychological assessments may not formally identify these mild, but nonetheless functionally disruptive changes [12].” In contrast, self-report methods may be more sensitive to identify subtle changes, “because self-report taps a patient’s self-knowledge of their previous ability, whereas neuropsychological testing usually approximates premorbid functioning by using test-based norms [37]”. In particular, self-reported cognitive measures also require substantially fewer resources than do formal neurocognitive tests, due to the lack of practice effects and clinical adaptability [16, 37]. While self-reported cognitive measures have several important strengths in research settings, future studies should utilise both subjective and objective neuropsychological assessments to quantify the prevalence, severity, and impact of CRCI in the Chinese gynaecological cancer population, as few studies have been conducted to date on Chinese cancer survivors.

This study found that patients after chemotherapy reported the lowest level of small-worldness index and global and local network efficiency, compared with age-matched non-cancer controls. Research evidence shows that disrupted structural networks have been demonstrated to have detrimental effects on cognitive functioning [10, 18, 22, 38]. Global and local network efficiency has been demonstrated to be important for cognitive functioning, as global efficiency plays a key role in how information may be efficiently exchanged across the entire brain network [39]. In contract, local network efficiency measures the average of local subgraphs in a network and indicates how tolerant a network is to local failures [40]. Regarding the associations between structural network properties and subjective cognitive impairment, this study found that higher values of small-worldness index and shorter characteristic path length were related to higher FACT-Cog total scores (i.e. better cognitive functioning). Study findings reveal that primary cancer treatment can result in a more random organisation of brain network changes, which contributed to reducing brain functional specificity and segregation, with implications for cognitive functioning [10].

Limitations of the cross-sectional study design were the inability to explore the course of CRCI over time; additionally, the study could not provide causal inferences for factors associated with subjective cognitive impairment. Thus, future prospective cohort studies should be conducted to explore the causal associations between CRCI and clinical factors, psychosocial variables, and brain networks. In addition, this study did not collect details about patients’ chemotherapy regimens. Hence, this study cannot discuss which chemotherapeutic agents may influence cognition. Finally, heterogeneity of clinical variables, such as cancer type, disease stage, and treatment modalities may have created sampling bias, which limits the generalisability of the results. While the present study has limitations that need to be addressed in future studies, its findings are important to communicate to patients and clinicians alike, especially due to the increasing level of concern about subjective cognitive impairment following cancer treatment.

## Conclusion

When compared with non-cancer controls, a considerable proportion of gynaecological cancer survivors may exhibit CRCI. After cancer treatment, 40.51% of Chinese gynaecological cancer patients perceived cognitive impairment. Moreover, cognitive impairment occurred not only in patients who had received chemotherapy, but also in approximately 10% of patients who were treated using surgery only. Lower cognitive functioning was associated with unemployment, receipt of chemotherapy, and depressive symptoms. This study provides the first evidence of brain structural network alteration in gynaecological cancer patients post-treatment, and offers novel insights regarding the neurobiological change mechanisms of CRCI in gynaecological cancer patients. Primary cancer treatment can result in a more random organisation of structural brain networks, which may reduce brain functional specificity and segregation and have implications for cognitive impairment. Future prospective and longitudinal studies are required to build upon the study findings in order to assess potentially relevant clinical and psychosocial variables and brain network measures, so as to more accurately understand the specific risk factors related to CRCI in the gynaecological cancer population. Such knowledge could inform the development of appropriate treatment and rehabilitation efforts to ameliorate CRCI in gynaecological cancer survivors.

## Abbreviations

BFI: Brief Fatigue Inventory
CRCI: Cancer-Related Cognitive Impairment
DTI: Diffusion Tensor Imaging
EPI: Echo-Planar Imaging
FA: Fractional Anisotropy
FACT-Cog: Functional Assessment of Cancer Therapy-Cognitive Function
GRETNA: Graph Theoretical Network Analysis
HADS: Hospital Anxiety and Depression Scale
MNI: Montreal Neurological Institute
MRI: Magnetic Resonance Imaging
PANADA: Pipeline Toolbox for Analysing Brain Diffusion Images
